# Selective Inhibition of the Immunoproteasome β5i Prevents PTEN Degradation and Attenuates Cardiac Hypertrophy

**DOI:** 10.3389/fphar.2020.00885

**Published:** 2020-06-12

**Authors:** Xin Xie, Hong-Xia Wang, Nan Li, Ya-Wen Deng, Hai-Lian Bi, Yun-Long Zhang, Yun-Long Xia, Hui-Hua Li

**Affiliations:** ^1^Department of Cardiology, Institute of Cardiovascular Diseases, First Affiliated Hospital of Dalian Medical University, Dalian, China; ^2^Department of Emergency Medicine, Beijing Key Laboratory of Cardiopulmonary Cerebral Resuscitation, Beijing Chaoyang Hospital of Capital Medical University, Beijing, China

**Keywords:** β5i, cardiac hypertrophy, immunoproteasome, PR-957, phosphatase and tensin homolog on chromosome ten

## Abstract

Cardiac hypertrophy without appropriate treatment eventually progresses to heart failure. Our recent data demonstrated that the immunoproteasome subunit β5i promotes cardiac hypertrophy. However, whether β5i is a promising therapeutic target for treating hypertrophic remodeling remains unknown. Here, we investigated the effects of PR-957, a β5i-specific inhibitor, on angiotensin II (Ang II)–induced hypertrophic remodeling in the murine heart. The infusion of Ang II increased immunoproteasome chymotrypsin-like activity and β5i catalytic subunit expression in the heart, whereas PR-957 treatment fully blocked the enhanced immunoproteasome activity caused by Ang II. Moreover, the administration of PR-957 significantly suppressed Ang II–induced cardiac hypertrophy, fibrosis, and inflammation. Mechanistically, PR-957 treatment inhibited phosphatase and tensin homolog on chromosome ten (PTEN) degradation, thereby inhibiting multiple signals including AKT/mTOR, ERK1/2, transforming growth factor-β, and IKB/NF-kB. Furthermore, PTEN blocking by its specific inhibitor VO-OHpic markedly attenuated the inhibitory effect of PR-957 on Ang II–induced cardiac hypertrophy in mice. We conclude that PR-957 blocks PTEN degradation and activates its downstream mediators, thereby attenuating Ang II–induced cardiac hypertrophy. These findings highlight that PR-957 may be a potential therapeutic agent for Ang II-induced hypertrophic remodeling.

## Introduction

Pathological cardiac hypertrophy frequently progresses to heart failure, which is associated with high mortality but without adaptive treatment. Besides increasing cell size, cardiac hypertrophy involves increased protein synthesis controlled by the PI3K/AKT/mTOR pathway. AKT is a key regulator of cell growth and generally works with several downstream effectors such as mammalian target of rapamycin (mTOR), GSK-3β, and FOXOs, ultimately resulting in cardiac hypertrophy (Condorelli et al., 2002; Nagoshi et al., 2005). Phosphatase and tensin homolog on chromosome ten (PTEN) is a negative regulator of AKT signaling and reportedly suppresses cardiac hypertrophy (Schwartzbauer and Robbins, 2001). PTEN inhibition in mice promotes cardiac hypertrophy and a marked decrease in cardiac contractility (Roe et al., 2015). Interestingly, our and other studies revealed that the ubiquitin-proteasome system (UPS) is responsible for PTEN degradation, but the precise mechanism requires further investigation (Wu et al., 2003; Chen et al., 2019).

The UPS plays an important role in controlling protein degradation in the cardiac hypertrophy process. Blockage of the UPS by proteasome inhibitors alleviates cardiac hypertrophy and improves cardiac function (Depre et al., 2006; Cacciapuoti, 2014). The standard subunits including β1, β2, and β5 form the essential ring structure of proteasome and contribute to caspase-like, trypsin-like, and chymotrypsin-like proteolytic activities, respectively (Angeles et al., 2012). Interestingly, stimulation of cells by inflammatory cytokines such as interferon-γ (IFN-γ) can induce the expression of three alternative catalytic β-subunits including β1i (LMP2), β2i (MECL-1 or LMP10), and β5i (LMP7) instead of three standard catalytic β-subunits to form immunoproteasome (Murata et al., 2009). Importantly, β5i is the most important catalytic subunit since it is preferentially incorporated into mature proteasomes and essential to maturation of the 20S proteasome (Hirano et al., 2008; Angeles et al., 2012). Our previous recent data revealed that β5i deletion alleviates pressure overload–induced cardiac hypertrophy and fibrosis in a murine model *via* activating autophagy; conversely, β5i overexpression in transgenic mice aggravates this effect (Xie et al., 2019). Further, genetic ablation and pharmacological inhibition of β5i reduces deoxycorticosterone-acetate (DOCA) salt–induced cardiac remodeling in mice (Cao et al., 2019). Furthermore, β5i deletion decreases the incidence of atrial fibrillation (AF) that associated with inhibition of the inflammatory response, oxidative stress, and fibrosis in the atria of angiotensin II (Ang II)-infused mice (Li et al., 2019). These results suggest that β5i has a critical effect on cardiovascular diseases and that developing drugs to inhibit β5i and its responsible chymotrypsin-like activity would become a novel therapeutic strategy to treat hypertrophic cardiac diseases. PR-957 (also known as ONX-0914) is a potent and selective inhibitor of immunoproteasome subunit β5i (Kisselev and Groettrup, 2014). However, its effects on Ang II–induced cardiac hypertrophy and the involved molecular mechanisms remain unclear.

Here, we report the inhibitory effects of PR-957 on Ang II–mediated cardiac hypertrophy, fibrosis, and inflammation. We further provide new evidence that PR-957 administration inhibits proteasome chymotrypsin-like activity, preventing PTEN degradation and leading to subsequent attenuation of cardiac hypertrophic remodeling. Overall, these results suggest that PR-957 is a novel effective candidate drug for treatment of Ang II-induced cardiac hypertrophic remodeling.

## Materials and Methods

### Animals and Treatments

All mice were kept clean and dry in a comfortable environment and allowed to eat food and drink water freely at 20–25°C. Male 10–12-week-old wild-type C57BL/6 mice were anesthetized with isoflurane gas and osmotic mini-pumps (Alzet, Cupertino, CA, USA) containing saline or Ang II (1,000 ng/kg/min) was implanted under the back skin; the saline or Ang II release lasted for 14 days as described previously (Li et al., 2015; Wang et al., 2018). Before the saline or Ang II infusion, the mice received an intraperitoneal injection of PR-957 (12 mg/kg) or saline three times per week. An intraperitoneal injection of VO-OHpic (10 mg/kg/day), a PTEN inhibitor, was performed on mice in the presence or absence of PR-957 for 2 weeks before the infusion. Treatment of mice with PR-957 or VO-OHpic at those dosages significantly blocked chymotrypsin-like or PTEN activity, respectively, as described previously (Althof et al., 2018; Chen et al., 2019). Untreated age-matched mice were used as controls. All experiments performed on the mice were approved by the Animal Care and Use Committee of Dalian Medical University (authorization number YJ-KY-SB-2019-75).

### Reagents and Antibodies

Angiotensin II was purchased from Aladdin (Beijing, China). PR-957, VO-OHpic, cycloheximide, and wheat germ agglutinin (WGA) were obtained from Sigma-Aldrich (St. Louis, MO, USA). Antibodies against transforming growth factor-β1 (TGF-β1), β5i, phospho-IKBα, IKBα, and α-actinin were obtained from Abcam (Cambridge, UK). Antibodies against β1, β2, β5, β1i, β2i, rabbit anti-goat, and goat anti-rabbit and goat anti-mouse secondary antibody were purchased from the Proteintech (Wuhan, China). Antibodies against PTEN, phospho-AKT, AKT, phospho-mTOR, mTOR, phospho-p65, p65, phospho-ERK, ERK, and glyceraldehyde-3-phosphate dehydrogenase (GAPDH) were purchased from Cell Signaling Technology (Boston, MA, USA). Antibodies against F4/80 were purchased from BioLegend (USA). A cell-based proteasome assay kit was purchased from Promega Bioscience (Madison, WI, USA), hematoxylin and eosin (H&E) assay kit and Masson’s trichrome assay kit were purchased from Beyotime Biotechnology (Shanghai, China).

### Echocardiography

All mice were anesthetized with isoflurane gas and the heart rate maintained at 450–550 beats per minute. The cardiac functions of the mice were determined by transthoracic echocardiography after Ang II infusion and PR-957 administration by a 30-MHz transducer (Vevo 1100 system; VisualSonics, Toronto, Ontario, Canada) as described previously (Li et al., 2015). The left atrial diameter, left ventricular internal diameter (LVID) at diastole and systole, left ventricular anterior wall (LVAW) thickness at diastole and systole, left ventricular posterior wall (LVPW) thickness at diastole and systole, left ventricular ejection fraction (EF%), and left ventricular fractional shortening (FS%) were analyzed on the M-mode tracings and the average was obtained from at least 3 separate cardiac cycles. EF% and FS% were calculated as follows: 100×([LVEDV−LVESV]/LVEDV) (%) and 100×([LVDd−LVDs]/LVDd) (%).

### Histopathological Analysis

The heart tissues were fixed in formalin for 48 h at room temperature, embedded in paraffin, and then cut into 5-μm serial sections. H&E staining was performed to examine cardiac inflammation according to a standard protocol (Xie et al., 2019). To determine the extent of fibrosis in the heart tissues, the sections were stained with Masson’s trichrome (Sigma-Aldrich) according to the manufacturer’s procedures as described (Xie et al., 2019). Three or four slices from each mouse were quantified and a total of 20 slices from five mice in each group were analyzed. The areas of cardiac fibrosis were automatically identified by Image-Pro Plus 3.0 software (Nikon, Japan). To examine the cross-sectional area of myocytes, sections were stained with 50 μg/ml of rhodamine-labeled WGA for 60 min. Digital images were taken at ×200 magniﬁcation of more than 20 random ﬁelds from each heart sample. Each cell area was calculated by measuring 150–200 cells per sample (Xie et al., 2019). All images were recorded using an Olympus BX51 microscope (Tokyo, Japan) and analyzed by ImagePro Plus software.

### Immunohistochemistry

Heart sections (5-μm thick) were immersed in xylene and different concentrations of ethanol, then put into 0.01 M citric acid–sodium citrate at 121°C for 25 min to recover the antigen. After being washed by phosphate-buffered saline (PBS) for 5 min, the sections were permeabilized in 0.1% Triton X-100 for 15 min and blocked with goat serum for 30 min at room temperature. F4/80 (1:1,000 dilution) antibody was added to cover the sections at 4°C overnight. After three washes, with PBS, biotinylated secondary antibody was added for 1 h to react with F4/80. Thereafter, the sections were stained by diaminobenzidine and photographs were taken digitally using a microscope.

### Measurement of Proteasome Activity

The proteins were extracted from fresh left ventricular (LV) tissues with Hepes buffer (50 mM, pH 7.5). Forty micrograms of protein from each sample was prepared to examine the activities of the proteasome as described (Xie et al., 2019). Briefly, 40-µl protein samples were added to a black 96-well plate and then reacted with 100 µl of Z-LLE-AMC (45 µM), Ac-RLR-AMC (40 µM), or Suc-LLVY–AMC (18 µM) to evaluate the caspase-like, trypsin-like, and chymotryptic-like activities. After the plate was incubated in the dark at 37°C for 30 min, the fluorescence intensity was measured with excitation at 380 nm and emission at 460 nm using a microplate reader (Tecan Infinite M1000 Pro).

### Western Blot Analysis

The heart tissues were quickly removed, washed in ice-cold PBS, and snap frozen immediately in liquid nitrogen. Total proteins were isolated from on ice with RIPA buffer containing protease phosphatase inhibitor cocktail as described (Xie et al., 2019). The protein concentration of each sample was determined by a bicinchoninic acid assay kit (Thermo Fisher Scientific, USA). Forty micrograms of proteins of each sample was added to the sodium dodecyl sulfate–polyacrylamide gel (15% for β1, β2, β5, β1i, β2i, and β5i; 10% for others) and then transferred onto polyvinylidene difluoride membranes. After blocking with 5% non-fat milk, the membrane was incubated with appropriate primary antibodies overnight at 4°C and incubated with second antibodies for 1 h at room temperature. The protein bands were detected by the chemiluminescence method and the signal intensities were analyzed with a Gel-Pro 4.5 Analyzer (Media Cybernetics, Rockville, MD, USA). The band intensity was quantified and normalized to GAPDH.

### Quantitative Real-Time Polymerase Chain Reaction Analysis

Total RNA was extracted from fresh LV tissues using TRIzol (Invitrogen, Carlsbad, CA, USA) according to the manufacturer’s protocol, and cDNA was generated using a Reverse Transcriptase Kit (Takara) followed by the SYBR-Green method. The following primers were used: ANF (forward: 5′-TCGTCTTGGCCTTTTGGCT-3′; reverse: 5′-TCCAGGTGGTCTAGCAGGTTCT-3′), β-MHC (forward: 5′-CGAGGCAAGCTCACGTATAC-3′; reverse: 5′-CTTG GCTTCTGTTTCCTCCT-3′), IL-1β (forward: 5′-CTCTGTGACTCGTGGGATGATG-3′; reverse: 5′-CCACTTGTTGGCTTATGTTCTGTC-3′), IL-6 (forward: 5′-TCTGCTCTGGTCTTCTGGAG-3′; reverse: 5′-TTGCTCTGAATGACTCTGGC-3′), Collagen I (forward: 5′-GAGTACTGGATCGACCCTAACCA-3′; reverse: 5′-GACGGCTGAGTAGGGAACACA-3′), and Collagen III (forward: 5′-TCCCCTGGAATCTGTGAATC-3′; reverse: 5′-TGAGTCGAATTGGGGAGAAT-3′). GAPDH was considered a house-keeping gene to which all target genes were normalized. Relative transcript levels of the target genes were calculated according to the formula: X = 2^−Δct^, while ΔCt = Ct_target_ − Ct_GAPDH_ and X is the fold changes in target gene.

### Statistical Analysis

Data are presented as mean ± SEM. For comparison of two groups and normal distribution, Student’s t-test was performed. For multiple group, significance was determined by using one-way analysis of variance (ANOVA) followed by Tukey’s post test. Values of *P* < 0.05 were considered statistically significant.

## Results

### PR-957 Reduces Chymotrypsin-Like Activity in Ang II-Infused Hearts

**Figure 1:**
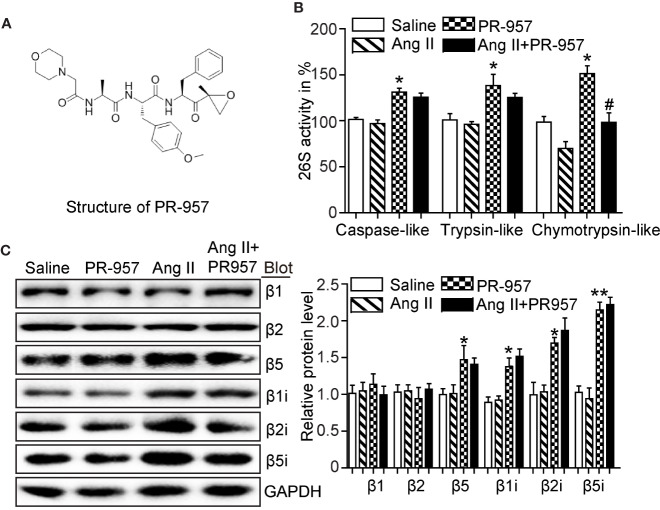
Effect of PR-957 on Ang II–upregulated proteasome subunit expressions and their corresponding proteasome activity. **(A)** The structure of PR-957. **(B)** Changes of proteasome activities in the LV tissues of Ang II–infused mice in the presence or absence of PR-957 for 2 weeks (n = 5). **(C)** Representative images of proteasome subunit expressions and the quantification of each protein band (n = 5). Data are expressed as mean ± SEM, and n represents the number of animals per group. *P < 0.05, **P < 0.01 vs. saline; ^#^*P* < 0.05 vs. Ang II.

PR-957 was a selective inhibitor of β5i, and its structure was shown (**Figure 1A**). To determine whether inhibition of β5i attenuates Ang II-induced cardiac remodeling, wild-type mice were co-treated with Ang II and β5i-specific inhibitor PR-957 for 2 weeks. We found that Ang II increased caspase-like, trypsin-like, and chymotrypsin-like activities compared with saline (**Figure 1B**). Ang II infusion also dramatically enhanced the protein levels of proteasome subunits including β5, β1i, β2i, and β5i versus saline-treated hearts (**Figure 1C**). Conversely, PR-957 treatment selectively inhibited Ang II–induced increases in chymotrypsin-like activity but had no effect on Ang II–upregulated proteasome subunit protein levels as well as caspase-like and trypsin-like activities (**Figures 1B, C**). These results suggest that PR-957 is specific inhibitor for chymotrypsin-like activity in the hearts.

### PR-957 Partly Reverses Ang II–Induced Cardiac Hypertrophy

**Figure 2:**
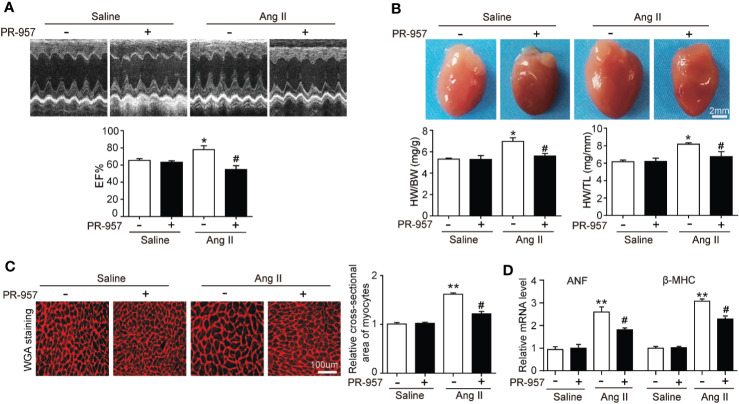
Administration of PR-957 reduces Ang II–induced cardiac hypertrophy *in vivo*. **(A)** Representative echocardiography images of Ang II–infused mice in the presence or absence of PR-957 for 2 weeks (top). Quantitative analysis of left ventricular (LV) ejection fraction (EF%, bottom, n = 5). **(B)** Representative images of heart size photographed with a stereomicroscope (top). Scale bar, 200 mm. Ratios of heart weight/body weight (HW/BW) and heart weight/tibial length (HW/TL, bottom, n = 5). **(C)** LV sections were stained with wheat germ agglutinin (WGA) to demarcate the cell boundaries (left). Scale bar, 100 μm. Quantification of the relative myocyte cross-sectional area (200 cells counted per heart) (right, n = 5). **(D)** Quantitative real-time polymerase chain reaction analysis of the mRNA levels of ANF and β-MHC (right, n = 5). Data are expressed as mean ± SEM, and n represents the number of animals per group. *P < 0.05, **P < 0.01 vs. saline; ^#^*P* < 0.05 vs. Ang II.

To further confirm the pathophysiological role of β5i in cardiac hypertrophy, the effects of PR-957, the selective inhibitor of β5i, on Ang II–induced hypertrophic hearts were examined. Echocardiographic measurements showed that Ang II infusion for 2 weeks resulted in adaptive increase of cardiac performance as indicated by EF% (**Figure 2A**) accompanied with increased LVAW and LVPW during systole and diastole versus the saline group, whereas this effect was dramatically attenuated by PR-957 (**Figure 2A** and **Table 1**). Moreover, Ang II infusion significantly promoted cardiac hypertrophy as evidenced by higher heart weight/body weight (HW/BW) ratio and cross-sectional area of myocytes in wild-type mice. In contrast, treatment with PR-957 markedly reduced Ang II–induced hypertrophic response (**Figures 2B, C**). Furthermore, qPCR analysis showed that the increased mRNA expression of hypertrophic markers such as ANF and β-MHC were significantly decreased in PR-957 and Ang II–co-treated mice compared with the Ang II–infused only mice (**Figure 2D**). There was no significant difference in these parameters between PR-957 and control groups after saline treatment (**Figures 2A–D** and **Table 1**).

**Table 1:** Echocardiographic Analysis in wild type mice after Ang II infusion and PR-957 treatment.

Parameter	Saline	PR-957	Ang II	AngII+PR-957
HR(bpm)	545 ± 3.5	538 ± 4.8	536 ± 6.7	544 ± 4.7
LV Mass(AW)(mg)	83.59 ± 3.86	84.45 ± 10.33	97.35 ± 6.36*	82.18 ± 4.46^#^
LVID;d(mm)	4.11 ± 0.08	4.08 ± 0.2	4.17 ± 0.04*	3.97 ± 0.1
LVID;s(mm)	2.64 ± 0.09	2.66 ± 0.15	2.37 ± 0.05*	2.75 ± 0.12
LVPW;d(mm)	0.68 ± 0.03	0.72 ± 0.03	0.98 ± 0.07*	0.65 ± 0.01^#^
LVPW;s(mm)	1.05 ± 0.04	1.05 ± 0.01	1.25 ± 0.08*	0.94 ± 0.05^#^
LVAW;d(mm)	0.63 ± 0.02	0.68 ± 0.03	0.81 ± 0.02*	0.61 ± 0.03^#^
LVAW;s(mm)	0.87 ± 0.02	0.85 ± 0.04	1.12 ± 0.05*	0.81 ± 0.04^#^
FS(%)	25.29 ± 0.22	26.33 ± 2.15	28.37 ± 3.31	24.55 ± 1.46

Values are mean ± SEM, n(%).

HR, heart rate; LV Mass, left ventricular mass; LVID, left ventricular internal diameter; LVPW, left ventricular posterior wall; LVAW, anterior left ventricular wall. *P < 0.05 vs Saline; ^#^P < 0.05 vs Ang II.

### PR-957 Alleviates Ang II–Induced Cardiac Fibrosis and Inflammation

**Figure 3:**
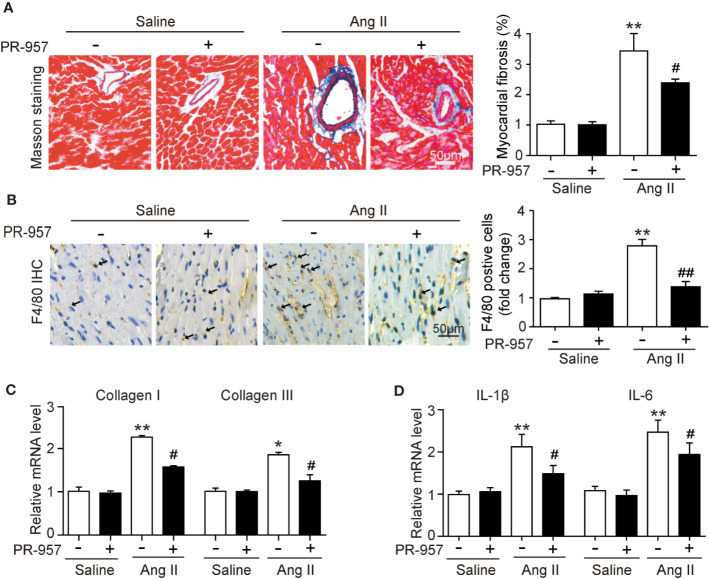
Administration of PR-957 attenuates Ang II–induced fibrosis and inflammation in mice. **(A)** Left ventricular sections were stained with Masson’s trichrome to detect fibrosis (left). Scale bar, 50 μm. Quantification of the relative fibrotic area (right, n = 5). **(B)** Immunohistochemical staining of cardiac macrophages with anti-F4/80 antibody (left). Scale bar, 50 μm. Quantification of the F4/80 positive cells (right, n = 5). **(C, D)** Quantitative real-time polymerase chain reaction analysis of the mRNA levels of collagen I, collagen III, interleukin (IL)-1β, and IL-6 (n = 5). **(D)** Data are expressed as mean ± SEM, and n represents the number of animals per group. *P < 0.05, **P < 0.01 vs. saline; ^#^*P* < 0.05 vs. Ang II ^##^*P* < 0.01 vs. Ang II.

F4/80 is specifically located in macrophages, and its appearance in the myocardium of the mice indicated the infiltration of inflammatory cells. To investigate whether PR-957 affects fibrosis and inflammation in Ang II–treated hearts, Masson’s trichrome staining and immunohistochemical detection for F4/80 were performed to examine the degrees of cardiac fibrosis and inflammation, respectively. While Ang II treatment caused a marked increase in cardiac fibrosis reflected by the increased collagen deposition labeled blue in the heart, PR-957 treatment significantly mitigated this response (**Figure 3A**). Immunohistochemistry using antibody to F4/80 showed that Ang II treatment significantly increased the infiltration of F4/80-positive macrophages in the heart compared with the saline group, and PR-957 alleviated this effect (**Figure 3B**). Parallel experiments with qRT-PCR analysis demonstrated that expressions of fibrotic markers collagen I and III, which are associated with the inflammatory mediators IL-1β and IL-6 in the heart, were dramatically increased in Ang II–infused hearts but was attenuated after PR-957 treatment (**Figures 3C, D**). Furthermore, no significant difference was observed between saline and PR-957 treatment alone (**Figures 3A–D**).

### PR-957 Inhibits Hypertrophic, Fibrotic, and Inflammatory Signaling Pathways in Ang II–Infused Heart

**Figure 4:**
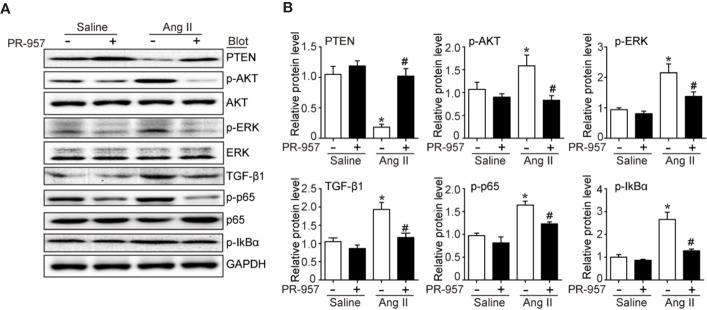
PR-957 inhibits activation of hypertrophic, fibrotic, and inflammatory signaling pathways in mice. **(A)** Western blot analysis of the protein levels of PTEN, p-AKT, AKT, p-ERK, ERK, TGF-β1, p-p65, p65, p-IkBα, IkBα, and GAPDH in Ang II–infused mice in the presence or absence of PR-957 for 2 weeks. **(B)** Quantification of each protein band (n = 5). Data are expressed as mean ± SEM, and n represents the number of animals per group. *P < 0.05 vs. saline; ^#^*P* < 0.05 vs. Ang II.

Since PTEN/PI3K/AKT, TGF-β1, and p-IkBα/NF-kB are key signaling pathways that mediate cardiac hypertrophy, fibrosis, and inflammation in the heart and atrial tissues (Li et al., 2018; Cao et al., 2019), we examined the effects of PR-957 on their expression in Ang II–infused hearts. Consistent with our previous findings (Li et al., 2018; Chen et al., 2019), we found that 2-week Ang II infusion significantly decreased PTEN protein level but increased protein levels of p-AKT, p-ERK, TGF-β1, p-p65, and p-IkBα compared with the saline control (**Figures 4A, B**). Conversely, these effects were partly reversed by PR-957, indicating that these signaling pathways were mediated by β5i in Ang II–induced cardiac hypertrophy (**Figures 4A, B**).

### PTEN Inhibitor Abolishes the Protective Effect of PR-957 on Cardiac Hypertrophic Remodeling in Mice

**Figure 5:**
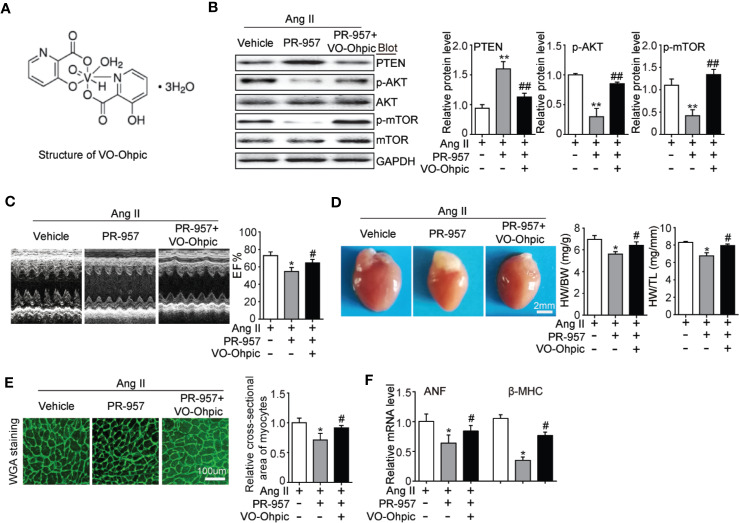
Blocking of phosphatase and tensin homolog on chromosome ten (PTEN) activity suppresses PR-957–mediated inhibitory effect on cardiac hypertrophy in mice. **(A)** The structure of VO-Ohpic. **(B)** Western blot analysis of PTEN, phospho-AKT, AKT, phospho-mTOR, mTOR, and GAPDH in LV tissues from mice after Ang II infusion in the presence or absence of PR-957 (12 mg/kg BW) together with VO-OHpic (10 mg/kg BW) (left) and quantification of each protein band (left, n = 5). **(C)** Representative echocardiography images of left ventricular chamber (top), and measurement of left ventricular ejection fraction (LV EF%, bottom). **(D)** Representative images of heart size (top) and ratios of heart weight/body weight (HW/BW) and heart weight/tibial length (HW/TL) (bottom, n = 5). **(E)** LV sections were stained with WGA to demarcate cell boundaries (left). Quantification of the relative myocyte cross-sectional area (200 cells counted per heart) (right, n = 5). **(F)** Quantitative real-time polymerase chain reaction analysis of the mRNA expression of ANF (right, n = 5). Data are expressed as mean ± SEM, and n represents the number of animals per group. *P < 0.05, **P < 0.01 vs. Ang II; ^#^*P* < 0.05, ^##^*P* < 0.01 vs. Ang II + PR-957.

VO-OHpic was an inhibitor of PTEN and its chemical structure was shown in **Figure 5A**. To further verify the involvement of PTEN in the PR-957–mediated inhibitory effects on cardiac hypertrophy, the PTEN inhibitor VO-OHpic was used in Ang II– and PR-957–treated mice. We found that PR-957 upregulated PTEN protein expression and inhibited AKT/mTOR signaling in the Ang II–infused heart; these changes in signaling pathway were reversed by VO-OHpic (**Figure 5B**). Moreover, PR-957 markedly improved Ang II–induced cardiac contractile dysfunction as reflected by EF%, and reduced cardiac hypertrophy as indicated by decreases in heart size, HW/BW and HW/TL ratios, cross-sectional area of myocytes and ANF expression (**Figures 5B–E**). The changes of other echocardiographic parameters for each group were shown in **Table 2**. Conversely, VO-OHpic treatment successfully reversed the cardioprotective effects of PR-957 (**Figures 5B–E** and **Table 2**). Taken together, these results suggest that PR-957 blocked Ang II–induced cardiac hypertrophy by enhancing PTEN stability.

**Table 2:** Echocardiographic Analysis in Ang II-infused mice after PR-957 and VO-Ohpic treatment.

Parameter	Ang II	AngII+PR-957	AngII+PR-957+VO-Ophpic
HR(bpm)	525 ± 12.5	544 ± 4.7	537 ± 15.8
LV Mass(AW)(mg)	108.7 ± 9.3	82.18 ± 4.46*	91.04 ± 3.51^#^
LVID;d(mm)	4.36 ± 0.13	4.17 ± 0.1	4.18 ± 0.05
LVID;s(mm)	3.2 ± 0.15	2.85 ± 0.12	2.94 ± 0.1
LVPW;d(mm)	0.85 ± 0.05	0.65 ± 0.01*	0.78 ± 0.01^#^
LVPW;s(mm)	1.15 ± 0.06	0.84 ± 0.05*	0.95 ± 0.03^#^
LVAW;d(mm)	0.86 ± 0.04	0.61 ± 0.03*	0.74 ± 0.03^#^
LVAW;s(mm)	1.11 ± 0.04	0.81 ± 0.04*	0.95 ± 0.04^#^
FS(%)	27.47 ± 4.11	24.54 ± 1.95	26.66 ± 1.87

Values are mean ± SEM, n(%).

HR, heart rate; LV Mass, left ventricular mass; LVID, left ventricular internal diameter; LVPD, left ventricular posterior wall; LVAW, anterior left ventricular wall. *P < 0.05 vs Saline; ^#^P < 0.05 vs Ang II+PR-957.

## Discussion

**Figure 6:**
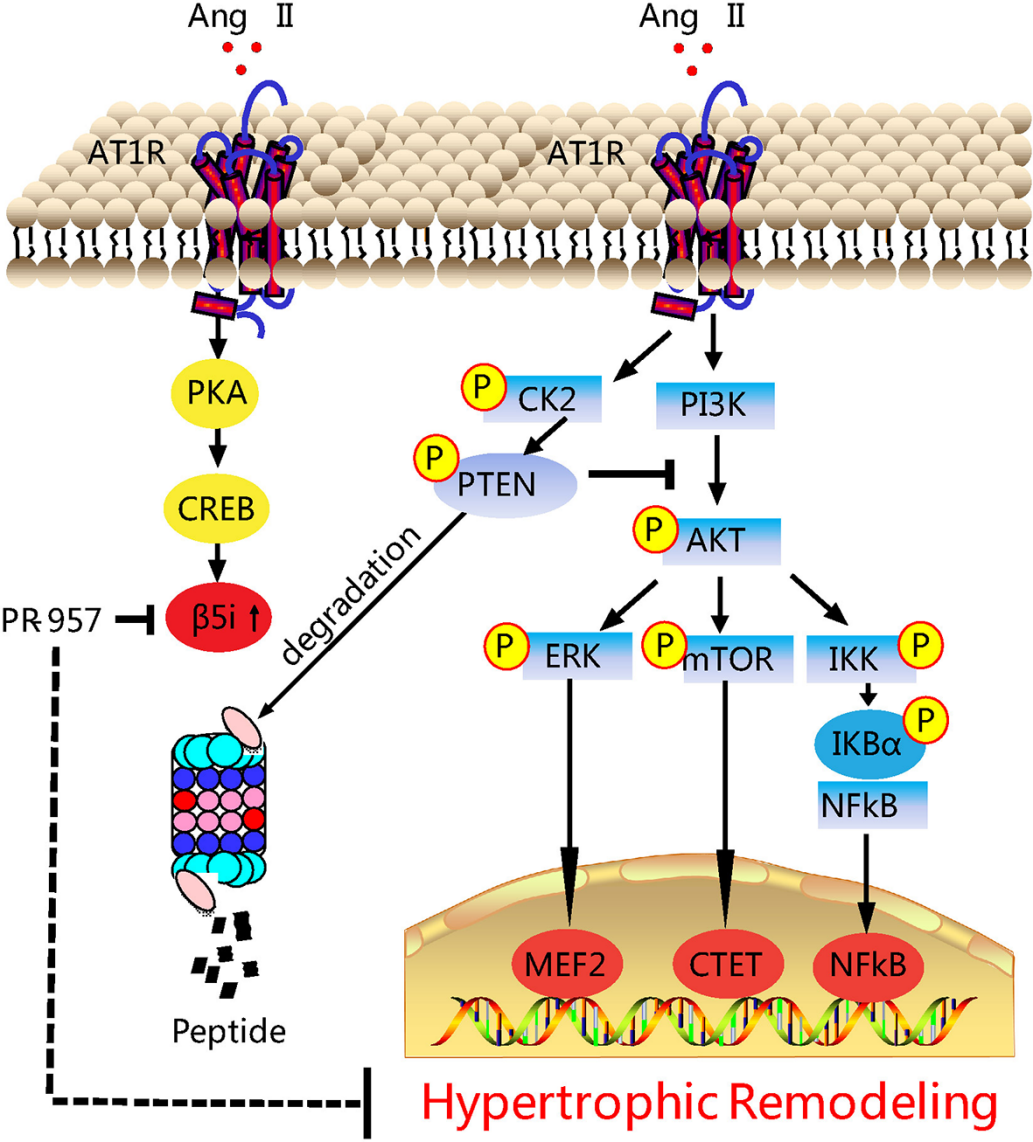
Working model of PR-957–mediated cardioprotection against Ang II–induced cardiac hypertrophy. Ang II upregulates β5i protein level and its corresponding chymotrypsin-like activity, which in turn promotes degradation of phosphatase and tensin homolog on chromosome ten (PTEN), leading to the activation of AKT/mTOR, TGF-β/Smad2/3, and p-IkBα/NF-KB, thereby resulting in cardiac hypertrophy, fibrosis, and inflammation. Conversely, the inhibition of β5i by PR-957 markedly reverses these effects. Casein Kinase II (CK2); Phosphoinositide 3-kinase (PI3K); protein kinase A (PKA); AMP-responsive element binding protein (CREB); myocyte enhancer factor 2 (MEF2).

Our previous study revealed that the upregulation of β5i associated with enhanced chymotrypsin-like activity was correlated with hypertrophic remodeling (Xie et al., 2019). Considering the evidence that chemical chaperones can inhibit chymotrypsin-like activity, here we investigated the potential of PR-957 as a therapeutic drug in a mouse model of Ang II–induced cardiac hypertrophy. Our results showed that the administration of PR-957 specifically reduced chymotrypsin-like activity and blocked PTEN degradation, resulting in the prevention of hypertrophic remodeling including cardiac hypertrophy, fibrosis, and inflammation in mice after 2-week Ang II infusion (**Figure 6**). Therefore, the present study suggests that PR-957 may become a novel therapeutic drug to treat Ang II-induced cardiac hypertrophy.

The immunoproteasome reportedly plays a critical function in regulating inflammation and oxidative stress in different immune and inflammatory diseases (Angeles et al., 2012). Several recent investigations have demonstrated that the immunoproteasome catalytic subunits (such as β2i and β5i) and their corresponding proteasome activities were significantly upregulated in the hearts, and exert a critical role in cardiac hypertrophy induced by Ang II infusion or pressure overload (Li et al., 2018; Cao et al., 2019; Xie et al., 2019). Ang II infusion activates several signaling pathways including AKT/mTOR, TGF-β/Smad2/3, and NF-κB through Ang II type receptor (AT1R), causing hypertrophy, fibrosis, inﬂammation, and oxidative stress, finally leading to cardiac hypertrophy and AF (Li et al., 2018; Cao et al., 2019). Interestingly, knockout of β2i or β5i markedly abrogates these signaling pathways and cardiac hypertrophic remodeling in mice (Li et al., 2018; Cao et al., 2019). Therefore, drugs or chemicals inhibiting these immunoproteasome subunits may have anti-hypertrophic and anti-inflammatory effects. PR-957 is a selective inhibitor for β5i, which reportedly treats several inflammatory diseases and cancers, and has no significant cytotoxicity to animals and normal cells (Muchamuel et al., 2009; Chauhan et al., 2010; Ruschak et al., 2011). In mouse models of rheumatoid arthritis and lupus, PR-957 treatment reduces cellular infiltration, autoantibody levels, and cytokine production, including IL-2, IL-6, IL-23, IFN-γ, and TNF-α, effectively alleviating the inflammatory response (Muchamuel et al., 2009). Moreover, PR-957 is an effective treatment for bortezomib-resistant myelomas (Chauhan et al., 2010; Ruschak et al., 2011). The administration of PR-957 also attenuates dystrophic features in mouse models of Duchenne muscular dystrophy by reducing activated T cell infiltration, myofiber necrosis, and collagen deposition in skeletal muscle tissues (Farini et al., 2019). In cardiovascular diseases, PR-957 treatment reduces the cardiac infiltration of monocytes/macrophages and the production of proinflammatory cytokines and chemokines, thereby improving cardiac output and mortality during acute myocarditis (Althof et al., 2018). Furthermore, PR-957 attenuates isoproterenol-induced cardiac hypertrophy (Zhang et al., 2015) and prevents LV remodeling and heart failure after myocardial infarction (Day Sharlene et al., 2013). We recently found that PR-957 inhibits DOCA salt–induced cardiac remodeling in mice (Cao et al., 2019). However, its role in regulating Ang II–induced cardiac hypertrophy and the underlying mechanism remain unclear. In this study, we extend our previous findings and discovered that the administration of PR-957 attenuated cardiac hypertrophy, fibrosis, and inflammation and improved cardiac dysfunction in Ang II–infused mice (**Figures 2** and **3**). Together, our results indicated that β5i is an important regulator of cardiac remodeling in an Ang II–induced hypertensive model.

PTEN is a negative regulator of the PI3K/AKT/mTOR pathways, which are involved in the development of cardiac hypertrophy and dysfunction (Oudit and Penninger, 2009; Xu et al., 2014). Deletion of PTEN reportedly causes cardiac hypertrophy in mice through the activation of mTOR signaling and inhibition of autophagy. Conversely, the inhibition of mTOR by rapamycin abolishes this effect (Xu et al., 2014). Moreover, cardiomyocytes in the absence of PTEN expression contributed to the inhibition of Pink1-AMPK signaling and autophagy, resulting in cardiac dysfunction and hypertrophy (Roe et al., 2015). Previous studies suggested that PTEN degradation occurs through the UPS (Chen et al., 2019). We recently demonstrated that immunoproteasome subunits are responsible for regulating PTEN stability (Cai et al., 2008; Li et al., 2018). For example, knockout of β1i or β2i prevented PTEN degradation and subsequently inhibited AKT/IKK/NF-κB signal activation in ischemic hearts or AF (Cai et al., 2008; Li et al., 2018). Inhibition or knockout of β5i increases PTEN protein levels and inhibits activation of the AKT/mTOR, TGF-β/Smad2/3, and NF-kB pathways, leading to the attenuation of DOCA salt–induced cardiac remodeling (Cao et al., 2019). However, the role of PR-957 in regulating the stability of PTEN in Ang II–induced cardiac hypertrophy remains unclear. In this study, we demonstrated that PR-957 specifically reduced proteasome chymotrypsin-like activity and blocked PTEN degradation, inactivating AKT/mTOR, TGF-β1, and p-IkBα/NF-KB signaling, thereby reducing the extent of hypertrophic remodeling (**Figures 2**–**4**). In addition, the inhibition of PTEN by VO-OHpic reversed the PR-957–mediated inhibitory effect on cardiac hypertrophy (**Figure 5**), indicating that PR-957 attenuates Ang II–induced cardiac hypertrophy partially through enhancing PTEN protein level.

In conclusion, here we demonstrated the cardioprotective effects of PR-957 on Ang II–induced cardiac hypertrophic remodeling, possibly by blocking PTEN degradation and subsequent downstream mediators. These results suggest the potential of PR-957 as a therapeutic agent for Ang II-induced cardiac diseases. However, its beneficial effects on other cardiac hypertrophic models induced by pressure overload or high-salt diets remain unknown and require further investigation.

## Data Availability Statement

The raw data supporting the conclusions of this article will be made available by the authors, without undue reservation, to any qualified researcher.

## Ethics Statement

The animal study was reviewed and approved by The Animal Care and Use Committee of Dalian Medical University (authorization number YJ-KY-SB-2019-75).

## Author Contributions

XX, Y-WD, NL, Y-LZ, and H-LB performed research. XX, H-XW, and H-HL analyzed data. Y-LX and H-HL conceived and designed research. H-HL and XX wrote the paper.

## Funding

This work was supported by grants from the National Natural Science Foundation of China (81570207, 81330003, 81630009, and 81600315).

## Conflict of Interest

The authors declare that the research was conducted in the absence of any commercial or financial relationships that could be construed as a potential conflict of interest.
